# Congenitally corrected transposition of the great arteries and implantation of a leadless pacemaker: a case report

**DOI:** 10.1186/s13019-023-02257-7

**Published:** 2023-04-17

**Authors:** Qiao-yuan Li, Wen-long Dai, can-can Lin, Xu Liu, Cheng-jun Guo, Dong Jian-zeng

**Affiliations:** Department of Cardiology, Beijing Anzhen Hospital, Capital Medical University, No.2 Anzhen Road, Chaoyang District, Beijing, 100029 China

**Keywords:** Cardiology, Transposition of great arteries, Atrioventricular block, Leadless pacemaker

## Abstract

**Background:**

Congenitally corrected transposition of the great arteries (ccTGA) is a rare cardiac anomaly and can lead to abnormal electrical activity of the heart. The implant of a pacemaker in such patients is more complicated than conventional operations. This case report of an adult with ccTGA who had a leadless pacemaker implant will provide a reference for diagnosing and treating such patients.

**Case presentation:**

A 50-year-old male patient was admitted to hospital having experienced intermittent vision loss for a month. An electrocardiogram and Holter monitoring showed intermittent third-degree atrioventricular block, and echocardiography, cardiac computed tomography and cardiac magnetic resonance imaging confirmed a diagnosis of ccTGA. A leadless pacemaker was successfully implanted into the patient’s anatomical left ventricle, and the postoperative parameters were stable.

**Conclusion:**

Implanting a leadless pacemaker into a patient with a rare anatomical and electrophysiological abnormality, such as ccTGA, is feasible and efficacious, but preoperative imaging evaluation is of considerable importance.

## Background

Congenitally corrected transposition of the great arteries (ccTGA) is a rare congenital heart disease, accounting for < 1% of congenital heart diseases [1]. Its anatomical features are a heart that has a normal atrial position but abnormal atrioventricular (AV) connections. This means the positions of the left and right ventricles are reversed, so the anatomical left ventricle is on the right side and connected to the pulmonary artery, while the anatomical right ventricle is on the left side and connected to the aorta. These anatomical abnormalities can lead to abnormal cardiac electrophysiological function. Studies have shown that about 30% of patients with ccTGA have a complete AV block, which may be related to the anterior and superior transition of the AV node or the conduction abnormality below the AV node [2]. Therefore, pacemaker implantation and electrode positioning in such patients differ from the procedures performed in ordinary patients, and the operation is much more complex than conventional pacemaker implantation. Implantation of a leadless pacemaker in a subpulmonic left ventricle with fewer trabeculae is feasible but can be technically challenging, especially when there is dextrocardia. This paper reports the implantation of the latest generation leadless pacemaker in a patient with ccTGA to provide a reference for clinical diagnosis and treatment.

## Case presentation

### Chief complaint

A 50-year-old male patient was admitted to hospital on 16 November 2021 having suffered an intermittent loss of vision for a month. A dynamic electrocardiogram (ECG) taken at another hospital showed a high degree of AV block, with the most prolonged RR interval being 6.765 s, and an echocardiogram showed ccTGA.

### Electrocardiogram parameters

**Fig. 1:**
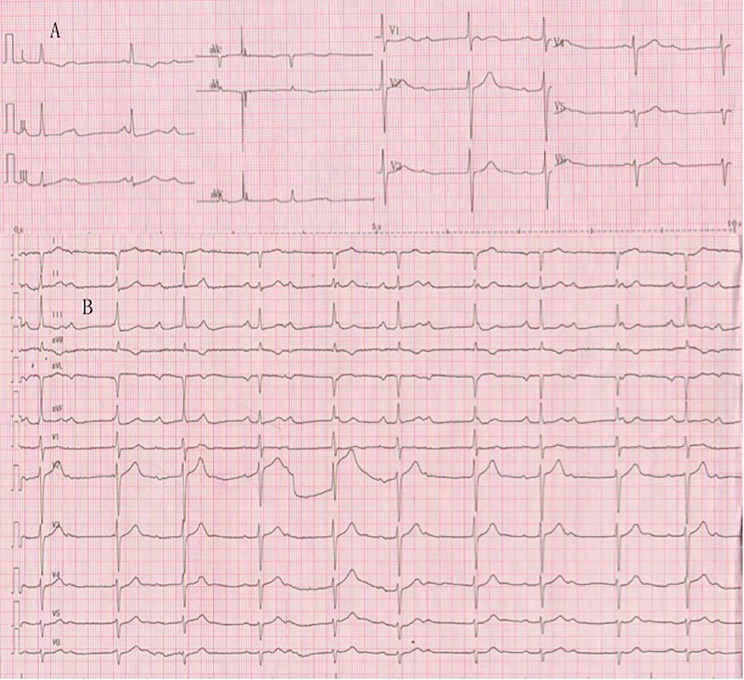
Electrocardiogram after admission (A: limb leads are connected; B: limb leads are reversed), it is a III° atrioventricular block, and the precordial leads are consistent with the characteristics of dextrorotatory heart

After admission, the ECG showed an intermittent third-degree AV block, Rs configuration in lead I, RS configuration in lead III and a gradually decreasing R wave amplitude in leads V1 to V5. The ECG after limb–lead reversal showed a QS pattern in lead I, an R pattern in lead III and a gradually decreasing R wave amplitude in leads V1 to V6 (Fig. 1).

### Image data

**Fig. 2:**
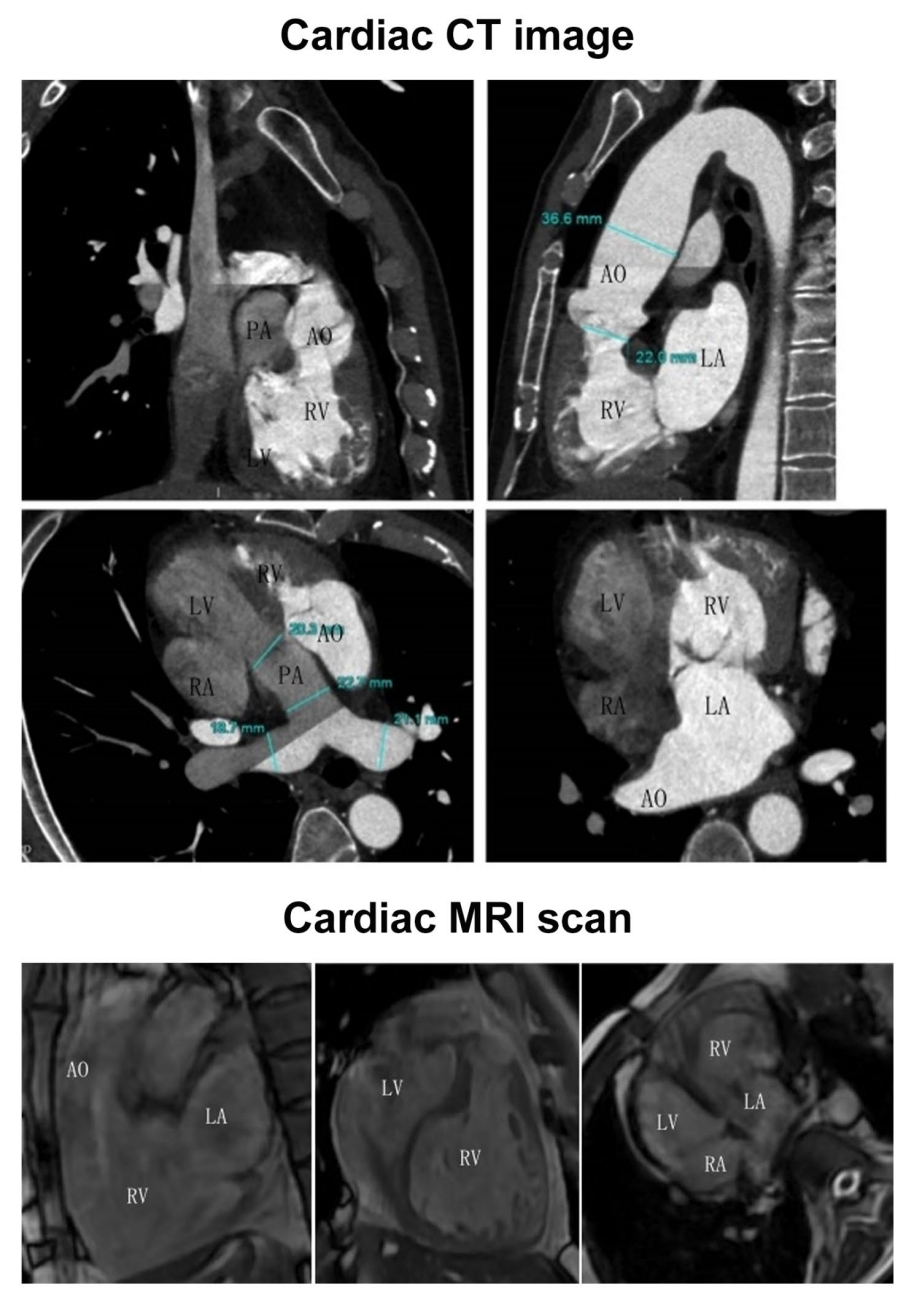
Cardiac CT and MRI image (PA = pulmonary artery, AO = aorta, LA = left atrium, RA = right atrium, LV = left ventricle, RV = right ventricle). Both CT and MRI images showed that the aorta was connected to the right ventricle and the pulmonary artery was connected to the left ventricle. The myocardium in the anatomical right ventricle was thicker than that in the anatomically normal right ventricle, and the trabecular myocardium in the anatomical left ventricle was less

Echocardiography showed a left ventricular ejection fraction of 0.55 and confirmed cardiac axis deviation and ccTGA, showing moderate tricuspid insufficiency, global enlargement of all four chambers, mild aortic regurgitation and mild pulmonary regurgitation. Cardiac computed tomography (CT) examination showed congenital heart disease, axis deviation, and function-corrected transposition of the great arteries (Fig. 2). A cardiac magnetic resonance imaging (MRI) scan showed that the aorta emerged from the right ventricle and the pulmonary artery was connected to the left ventricle (Fig. 2).

### Preoperative diagnosis and evaluation

There were several points to take into consideration before the implantation. First, the reason that a leadless pacemaker was suitable for this patient was that leadless pacing has a low anticipated pacing burden, especially in young patients, which keeps the number of necessary replacements low. Second, this patient had a low anticipated pacing burden because his symptoms occurred at rest at an intrinsic rate of 50 beats/min. In addition, the patient was young and had a normal body mass index and wished to have minimal body scarring.

### Intraoperative procedure and postoperative follow-up

**Fig. 3:**
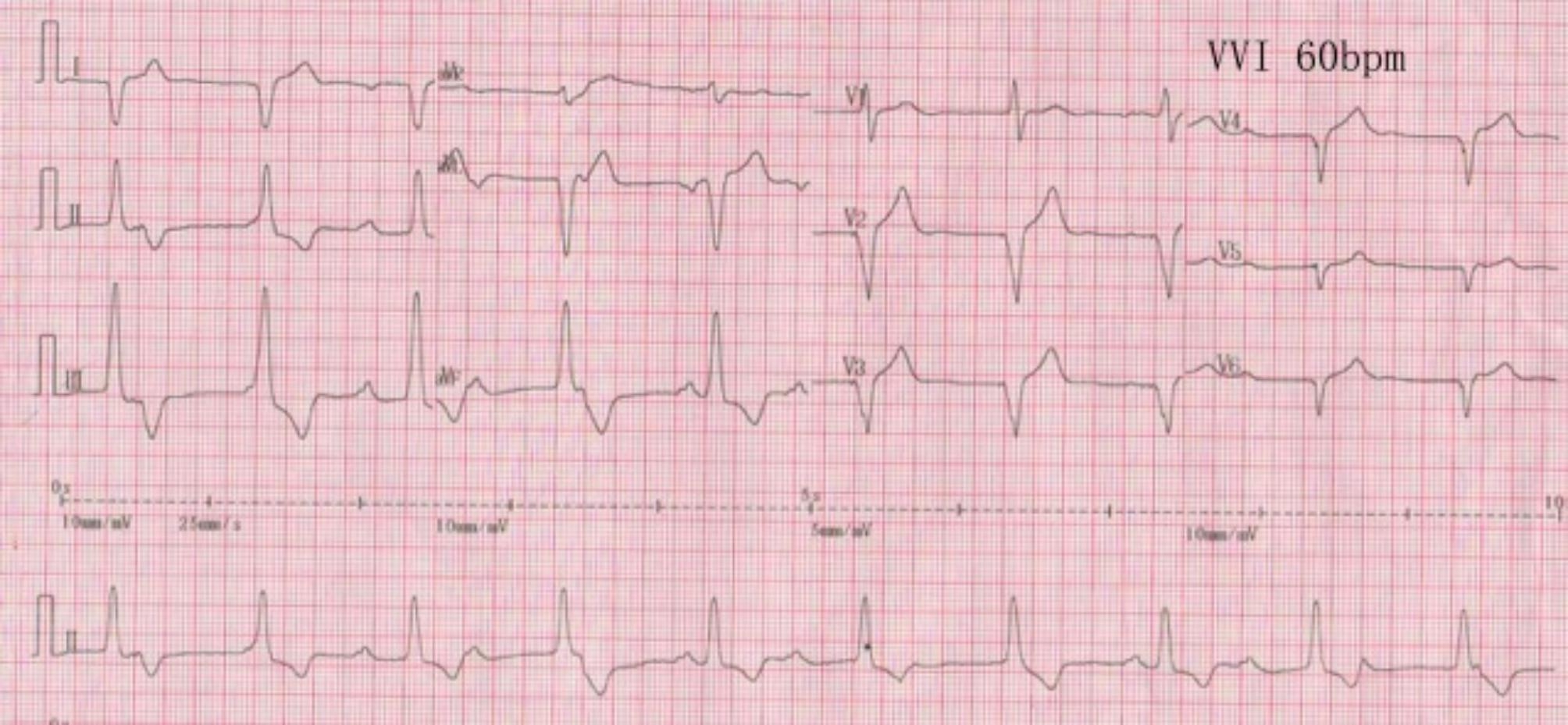
ECG at VVI 60 beats/min. The electrocardiogram shows that the main wave in leads II, III, and avF is directed upward, and the QRSd is about 120 ms, which is narrower than that of the apical pacing pattern

**Fig. 4:**
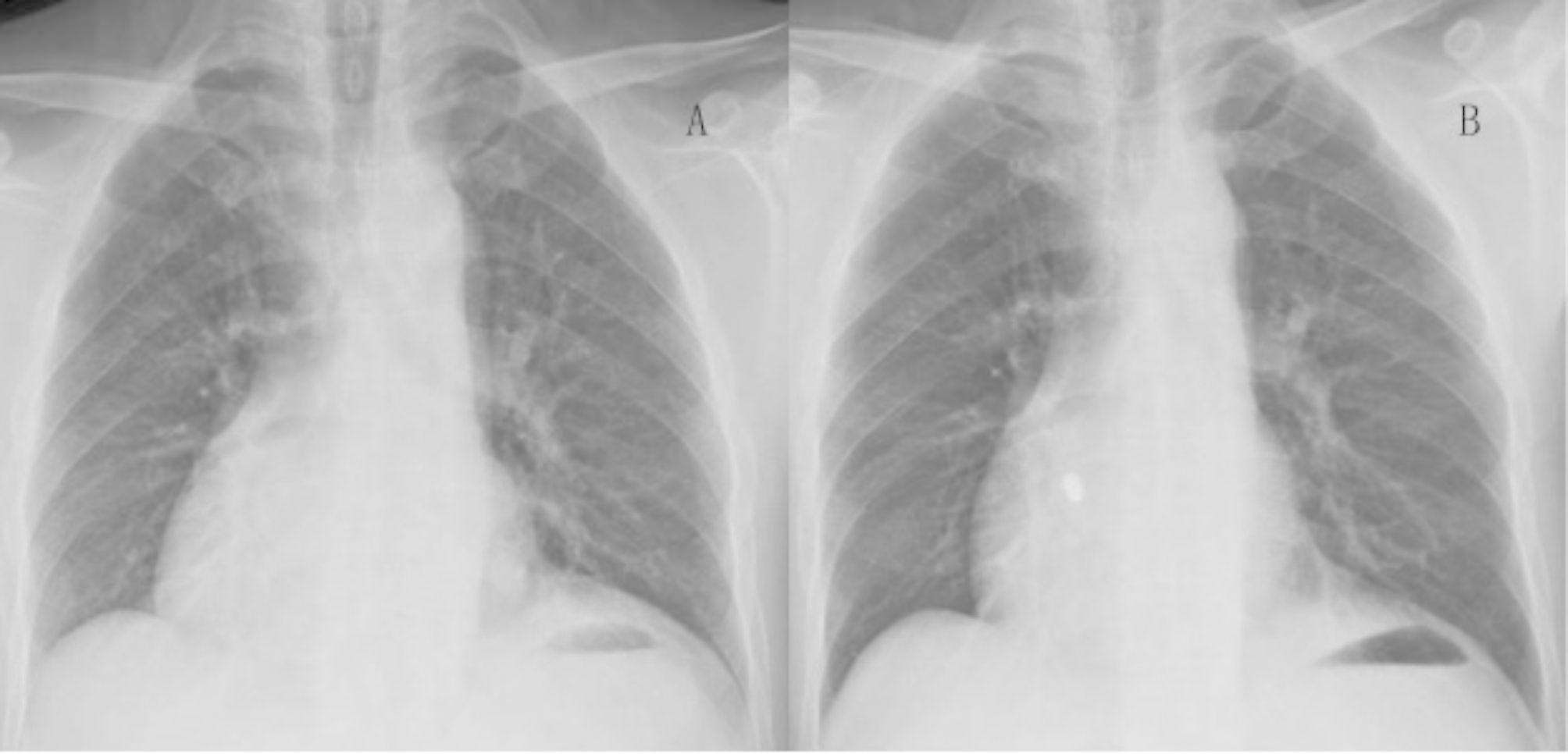
Comparison of preoperative and postoperative chest radiographs (A: preoperative chest radiograph; B: postoperative chest radiograph). The chest X-ray before discharge showed that the Micra position was fixed, and the cardiac shadow and costophrenic angle did not change significantly before and after procedure

The procedure took 45 min, and the fluoroscopy time used in the procedure was 20 min. Twenty-four hours after the operation, the threshold value of the leadless pacemaker was 0.88 V/0.24 ms, the sensed value was 8.6 mV and the impedance was 850 Ω. There were technical difficulties during implantation, which differed from traditional left ventricular implantation, due to the presence of ccTGA and dextrocardia. First, the catheter handle had to be pushed down instead of up, to ensure that the sheath could cross the AV flap to the left and enter the anatomical left ventricle when the handle’s big bend button was pressed, thus enabling the anatomical left ventricle of the patient to be evaluated using preoperative imaging. It was found that the pectinate muscle was not as developed as the anatomical right ventricle, and the delivery system was prone to slipping during implantation. This meant that it could not hold up the ventricular septum, and this affected the release and electrical parameters of the pacemaker. Postoperative pacing showed that the primary waves in leads II, III, and avF were upward, and some of the QRS intervals were more than 120 ms (Fig. 3). Postoperatively, the pacing threshold, R-wave amplitude and impedance were all excellent and stable. The chest X-ray before discharge showed that the Micra pacemaker position was fixed, and the cardiac shadow and costophrenic angle did not change significantly before and after the procedure (Fig. 4). The patient was observed for 24 h postoperatively under the pacing state of VVI 60 times/min and showed no symptoms of pacemaker syndrome, such as chest tightness and suffocation. The lower limit frequency of the pacemaker was lowered to 50 times/min, and when the patient did not complain of any obvious discomfort he was discharged on 24 November 2021, eight days after being admitted to hospital. After discharge, the patient’s activities returned to normal, and he could do physical exercise without any apparent distress. The 3-month follow-up after the procedure showed a pacemaker threshold of 0.88 V/0.24 ms, an R-amplitude of 10.4 mV and an impedance of 890 Ω. The electrical parameters at his 6-month follow-up were as follows: threshold, 0.75 V/0.24 ms; R-amplitude, 11.8 mV; and impedance, 860 Ω. Three months after the operation, the ventricular pacing (VP) was 34%, and the ECG showed sinus rhythm, and six months after the procedure, the VP was 32%, suggesting that most of the heart rhythms were generated by the patient and not the pacemaker.

## Discussion

Patients with a preoperative AV block are at increased risk of sudden cardiac death and therefore have more indications for pacemaker implantation than patients with structurally normal hearts. However, there are few case reports of the implantation of a leadless pacemaker in ccTGA patients [3, 4]. The latest adult congenital heart disease guidelines recommend pacemaker implantation in patients with tachy-brady syndrome, sinus or junctional bradycardia and impaired haemodynamics [5].

The preoperative ECG findings of the patient who received a leadless pacemaker implant reported in this paper were consistent with a third-degree AV block. His chest X-ray was consistent with dextrorotatory heart findings, and the cardiac CT and MRI examinations were consistent with ccTGA. After repeated consultations with the patient and his family, it was decided to implant a leadless pacemaker, which is very different from a traditional pacemaker, and there have been no previous case reports of a leadless pacemaker implanted in a ccTGA patient in China. After reviewing the relevant literature and refining the patient’s chest CT and cardiac MRI examinations, the anatomical relationship between the left and right atria, left and right ventricles and aorta and pulmonary arteries were defined. It was found that the myocardial trabecular content of the patient’s anatomical left ventricle was low, and there were difficulties in fixation and sheath slippage during the release of the leadless pacemaker during the procedure. Although implantation of a Micra pacemaker in a subpulmonic left ventricle with few trabeculae is feasible, it can be technically challenging, especially when there is dextrocardia. Therefore, this case can act as a guide for future leadless pacemaker implantation in patients with ccTGA.

Preoperative cardiac CT and intraoperative left ventriculography showed that the left atrium was connected to the aorta, the right ventricle to the tricuspid valve, the right atrium to the pulmonary artery and the left ventricle to the mitral valve. The pacemaker parameters were satisfactory and stable. The author’s experience is that if the patient’s cardiac anatomy is abnormal, the conventional leadless pacemaker operation involves deviating to the right, which is suitable for a trans-tricuspid valve operation of the left heart. However, this patient had a right-sided heart, which presented some difficulties. For the pacemaker to cross the mitral valve successfully, the operating rod has to be turned downward, and it can only point to the left AV valve when bent. This means that crossing and releasing the valve can be challenging for those not skilled at the procedure. At the same time, the patient’s preoperative and intraoperative images showed that the anatomical left ventricle had less trabecular myocardium, and it was difficult to fix the lead-free pacemaker. Consequently, the main focus during the operation was the fixability and suitable parameters.

## Conclusion

We reported a ccTGA patient with rare anatomical and electrophysiological abnormality who underwent leadless pacemaker implantation and aimed to provide evidence for this subgroup of patients. The importance of preoperative imaging evaluation for patients with rare anatomical and electrophysiological abnormalities cannot be understated. This case illustrates that the leadless pacemaker implant release position has to be controlled during the procedure to improve surgical efficiency and prevent complications.

## Data Availability

All data generated or analyzed during this study are included in this published article.
